# A Chemical Genetics Analysis of the Roles of Bypass Polymerase DinB and DNA Repair Protein AlkB in Processing *N* ^2^-Alkylguanine Lesions *In Vivo*

**DOI:** 10.1371/journal.pone.0094716

**Published:** 2014-04-14

**Authors:** Nidhi Shrivastav, Bogdan I. Fedeles, Deyu Li, James C. Delaney, Lauren E. Frick, James J. Foti, Graham C. Walker, John M. Essigmann

**Affiliations:** 1 Department of Biological Engineering, Massachusetts Institute of Technology, Cambridge, Massachusetts, United States of America; 2 Department of Chemistry, Massachusetts Institute of Technology, Cambridge, Massachusetts, United States of America; 3 Department of Biology, Massachusetts Institute of Technology, Cambridge, Massachusetts, United States of America; 4 Center for Environmental Health Sciences, Massachusetts Institute of Technology, Cambridge, Massachusetts, United States of America; University of Massachusetts Medical School, United States of America

## Abstract

DinB, the *E. coli* translesion synthesis polymerase, has been shown to bypass several *N* ^2^-alkylguanine adducts *in vitro*, including *N* ^2^-furfurylguanine, the structural analog of the DNA adduct formed by the antibacterial agent nitrofurazone. Recently, it was demonstrated that the Fe(II)- and α-ketoglutarate-dependent dioxygenase AlkB, a DNA repair enzyme, can dealkylate *in vitro* a series of *N* ^2^-alkyguanines, including *N* ^2^-furfurylguanine. The present study explored, head to head, the *in vivo* relative contributions of these two DNA maintenance pathways (replicative bypass vs. repair) as they processed a series of structurally varied, biologically relevant *N* ^2^-alkylguanine lesions: *N* ^2^-furfurylguanine (FF), 2-tetrahydrofuran-2-yl-methylguanine (HF), 2-methylguanine, and 2-ethylguanine. Each lesion was chemically synthesized and incorporated site-specifically into an M13 bacteriophage genome, which was then replicated in *E. coli* cells deficient or proficient for DinB and AlkB (4 strains in total). Biochemical tools were employed to analyze the relative replication efficiencies of the phage (a measure of the bypass efficiency of each lesion) and the base composition at the lesion site after replication (a measure of the mutagenesis profile of each lesion). The main findings were: 1) Among the lesions studied, the bulky FF and HF lesions proved to be strong replication blocks when introduced site-specifically on a single-stranded vector in DinB deficient cells. This toxic effect disappeared in the strains expressing physiological levels of DinB. 2) AlkB is known to repair *N* ^2^-alkylguanine lesions *in vitro*; however, the presence of AlkB showed no relief from the replication blocks induced by FF and HF *in vivo*. 3) The mutagenic properties of the entire series of *N* ^2^-alkyguanines adducts were investigated *in vivo* for the first time. None of the adducts were mutagenic under the conditions evaluated, regardless of the DinB or AlkB cellular status. Taken together, the data indicated that the cellular pathway to combat bulky *N* ^2^-alkylguanine DNA adducts was DinB-dependent lesion bypass.

## Introduction

**Figure 1:**
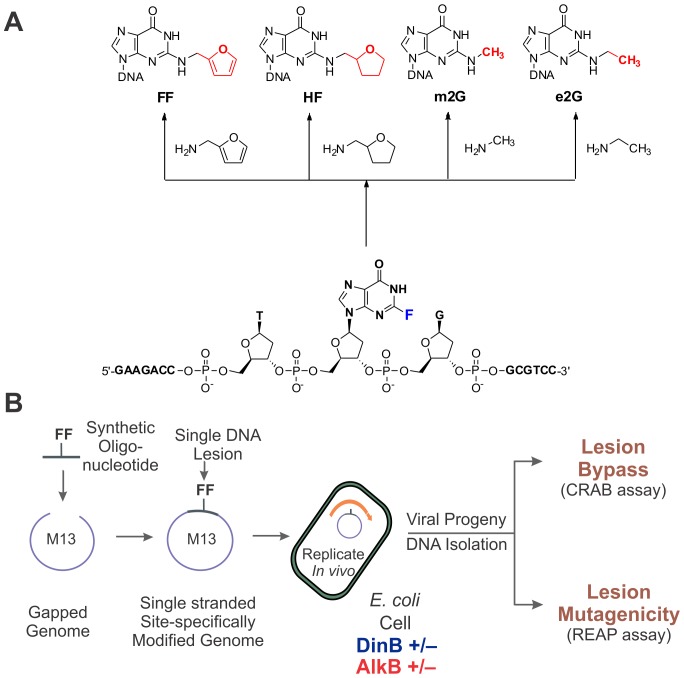
The structures of the *N* ^2^-alkylguanine lesions and the experimental strategy. (A) The synthesis of the 16-mer oligonucleotides containing *N* ^2^-alkylguanine lesions. The lesion-containing 16-mer oligonucleotides were synthesized by using the convertible nucleoside 2-fluoro-*O* ^6^-(trimethylsilylethyl)-2′deoxyinosine (bottom). The 2-fluoro atom (shown in blue) was then substituted with an amine group in parallel reactions with 2-fold molar excess of methyl-amine, ethyl-amine, furfurylamine and tetrahydrofurfuryl amine to yield m2G, e2G, FF and HF, respectively (top). See Materials and Methods for details. (B) Toxicity and mutagenicity assays. To determine the bypass and mutagenic properties *N* ^2^-alkyl guanine lesions *in vivo*, the oligonucleotide 16mers were synthesized and ligated into the genome of M13 bacteriophage, which was then replicated within *E. coli* cells lacking or expressing DinB or AlkB protein (4 strains in total). The viral progeny DNA was recovered and analyzed to determine two endpoints: 1) the relative reduction in progeny from lesion vs. a non-lesion competitor estimated the extent to which the *N* ^2^-alkylguanine lesions are blocks to DNA replication; 2) the base composition at the lesion site in the progeny indicated the extent and type of mutations induced by the studied lesions.

The genome is vulnerable to damage from exogenous and endogenous chemical reactions, including alkylation, oxidation, and deamination [1], [2]. Not surprisingly, several different lesion tolerance and repair pathways have evolved to deal with these types of DNA damage. DNA adduct bypass by translesion synthesis (TLS) polymerases allows for genome replication in the presence of DNA damage, while canonical DNA repair pathways, which include direct repair, base-excision repair, nucleotide-excision repair, non-homologous end joining and homologous recombination remove such damage prior to replication. A holistic approach for addressing the impact of such variables on lesion toxicity and mutagenicity is shown in Figure 1.

The present work explored the *in vivo* consequences (replication efficiency and fidelity) and genetic requirements (presence or absence of bypass polymerases or DNA repair enzymes) of four *N* ^2^-guanine DNA alkyl adducts: *N* ^2^-furfurylguanine (FF), 2-tetrahydrofuran-2-yl-methylguanine (HF), 2-methylguanine (m2G), and 2-ethylguanine (e2G) (Figure 1A). Previously, we have shown that the direct reversal DNA repair enzyme AlkB can repair these lesions *in vitro* [3]; the present study explored the relevance of AlkB repair of these lesions in living cells. Additionally, we have shown that both DinB and pol κ are capable of bypassing the FF lesion *in vitro* [4]; however, it remained unknown whether the same were true *in vivo*. Given these previous observations, both AlkB and DinB were selected as genetic variables for our *in vivo* chemical genetics study.

All of the *N* ^2^-alkylguanine lesions in this study are important biomarkers or structural mimics of exposure to known mutagens or carcinogens. The m2G adduct, the smallest alkyl adduct in the series, is a mimic of the imino or hydroxymethyl adducts formed by the reaction *N* ^2^-amino group of guanine with formaldehyde [5], [6]. Classified by the International Agency for Research on Cancer (IARC) as a human carcinogen [7], formaldehyde is an ubiquitous pollutant in vehicle exhaust and cigarette smoke and a common endogenous metabolism byproduct [7]. The m2G adduct can also form when cellular DNA is exposed to exogenous [5] or endogenous [8] methylating agents. The e2G DNA adduct is a well-established biomarker of exposure to acetaldehyde [9]–[14]. Acetaldehyde, classified as an animal carcinogen, and as a possible human carcinogen (group 2B) by IARC [15], is both an exogenous pollutant in cigarette smoke [16], [17] and an endogenous metabolite of ethanol [18]–[20]. The FF lesion is a mimic of the *N* ^2^-guanine adduct of nitrofurazone (NFZ) [21], a potent antibacterial agent commonly used for treating serious skin conditions (burns, grafts) [22], [23]. NFZ reduction metabolites have been shown to be mutagenic and carcinogenic in rodent models [22], [24] and to cause free radical damage, strand breaks, and *N* ^2^-dG adducts in DNA [25]–[27]. HF, the saturated analog of FF is included here to study the effect of aromaticity on bypass and repair of a bulky *N* ^2^-alkylguanine.

In *Escherichia coli*, the *dinB* gene encodes the Y family DNA polymerase pol IV (DinB) [28], [29], which is one of the three TLS polymerases that is part of the SOS pathway [30]. The *din*B gene was first identified as one of the *d*amage *in*ducible genes in *E. coli* [31]–[34], and it is the only Y-family DNA polymerase that is conserved across all domains of life (bacteria, eukaryotes, and archaea) [35], a result of selective constraints imposed on the encoding gene [36]. It is also present at a relatively high intracellular concentration of 250 molecules per cell, more than that of DNA pol III (10–30 molecules/cell) and on par with the level of the β-processivity clamp [37], [38]. Upon SOS induction, the concentration of DinB escalates to 2500 molecules per cell [39]. DinB is implicated in both the insertion and extension steps in the bypass of lesions that block replicative polymerases [40]. It may also have a role in alleviating the cytotoxicity of alkyl DNA adducts as demonstrated by Bjedov *et al*., who showed that DinB is essential for the survival of *ΔalkA Δtag* cells exposed to the alkylating agent methyl methanesulfonate [38]. *In vitro experiments* have shown that DinB can perform DNA synthesis, with efficiency and accuracy, across a variety of base modifications [29], such as FF [4] and N^2^-(1-carboxyethyl)-2′-deoxyguanosine (N^2^-CEdG) [41]. In vivo bypass is observed for site-specifically placed benzo[*a*]pyrene (BaP) lesions [42]–[45], and for lesions induced by chemical treatment of cells with 4-nitroquinoline-1-oxide (4-NQO) and NFZ [4] as well as incorporation of reactive oxygen species-derived dNTPs [46], [47]. The function of DinB (and also its human homolog pol κ) is of particular importance as cells are exposed to alkylating agents from both endogenous and exogenous sources, including cancer, inflammation and chemotherapy [2].

The AlkB enzyme is an Fe(II)- and α-ketoglutarate-dependent dioxygenase that repairs DNA alkyl lesions by a direct reversal of damage mechanism as part of the adaptive response in *E. coli* [48], [49]. Different homologs of AlkB exist in prokaryotic and eukaryotic species; nine such homologs exist in mammalian cells (ABH1-8 and FTO). The conservation of this enzyme across species underlies its importance as a defensive weapon in the cellular arsenal against DNA and RNA alkyl damage [50], [51]. AlkB can efficiently repair all N-methyl lesions on the Watson-Crick base pairing side of the four DNA bases [3]. These alkyl lesions include the simple adducts of 3-methylcytosine (m3C), 3-ethylcytosine, 1-methyladenine, 1-ethyladenine [52], 3-methylthymine, 1-methylguanine [53], as well as the recently reported 4-methylcytosine, and the four *N* ^2^-alkylguanines in the current study (m2G, e2G, FF and HF) [3]. Although AlkB can repair many of these lesions in a double-stranded DNA context, AlkB is much more efficient at repairing lesions in single-stranded DNA [54]–[56]. In the case of the *N* ^2^-alkylguanines, we have shown that *in vitro*, AlkB repairs these lesions only in single-stranded DNA; no repair was detected in double-stranded context [3].

In this work, we characterized the *in vivo* consequences of four *N* ^2^-dG lesions as a function of the bypass polymerase DinB and the DNA repair enzyme AlkB. Using genome site-specific mutagenesis methods [57], we inserted each of the four *N* ^2^-dG lesions at a specific location in single-stranded M13 phage DNA, which was then introduced into *E. coli* cell strains proficient or deficient for DinB and AlkB (a total of 4 possible strains). The mutation frequencies at the lesion site and bypass efficiencies across the lesions were measured *in vivo* using the restriction endonuclease and postlabeling (REAP) and competitive replication of adduct bypass (CRAB) assays, respectively [57].

## Materials and Methods

### Cell strains

All the *E. coli* strains used in this work contain the F′ episome, which enables infection by M13 phage. GW5100 strain was used for large scale preparation of M13 phage DNA; SCS110 (JM110, *endA1*) was used for amplification of progeny phage post-electroporation; NR9050 strain was used for double agar plating with X-gal for blue-clear detection of plaques. HK81 (as AB1157, but *nalA*) and HK82 (as AB1157, but *nalA alkB22*; AlkB-deficient) were the DinB^+^AlkB^+^ and DinB^+^AlkB^−^ strains used in the study.

P1 *vir* phage transduction was used to create HK83 (as HK81, but *dinB*-deficient) and HK84 (as HK82, but *dinB*-deficient). Briefly, recipient cells (HK81 and HK82) from 1 ml of overnight saturated cultures were resuspended in 500 µl LB containing 10 mM MgSO_4_ and 5 mM CaCl_2_. Approximately 100 µl of these solutions were mixed with 0, 25, 50, or 100 µl of P1 lysate containing a chloramphenicol resistance gene (*cam*) flanked by *frt* sequences designed for insertion at the *dinB* site. After 30 min incubation at 30°C, 100 µl of 1 M sodium citrate was added to stop the P1 infection. Following an additional incubation at 30°C for 1 h to allow expression of the chloramphenicol gene, the cells were plated on LB + chloramphenicol (10 µg/ml) plates. After an overnight incubation at 37°C, colonies were obtained, replated on LB + chloramphenicol plates and genotype confirmed by PCR.

### Oligonucleotides

All unmodified oligonucleotides and primers were obtained from Integrated DNA Technologies (IDT, Coralville, IA) unless specified otherwise. The lesion-containing 16mer oligonucleotides of the sequence 5′-GAAGACCTXGGCGTCC-3′ (where X denotes an *N* ^2^-alkylguanine lesion or controls) were synthesized using phosphoramidite solid-phase methods described before [3], [4], [58]. A convertible nucleoside, 2-fluoro-*O* ^6^-(trimethylsilylethyl)-2′deoxyinosine (ChemGenes, Wilmington, MA) was initially incorporated at the X position (Figure 1A). After hydrolysis from the resin and deprotection with 0.1 M NaOH for 8 h at 25°C, the oligonucleotides were desalted (SepPak, Millipore) and lyophilized. The 2-fluoro atom was then substituted with an amine group in parallel reactions with 2-fold molar excess of methyl-amine, ethyl-amine, furfurylamine and tetrahydrofurfuryl amine to yield m2G, e2G, FF and HF, respectively. The reactions were carried out in DMSO, in the presence of *N*,*N*-diisopropylethylamine (5X molar excess) at 60°C for 12 h. Finally, the trimethylsilylethyl group was removed with by treatment with an excess solution of 5% acetic acid at room temperature for 4 h. The deprotected oligonucleotides were purified by reversed-phase HPLC using an analytical column (Varian Microsorb-MV 100-5 C18 250×4.6 mm) at a flow of 1 ml/min and a gradient of 0 to 30% B over 60 min (A: 100 mM triethylammonium acetate; B: 100% acetonitrile).

Sixteen-mer oligonucleotides with the above sequence but with X = G, A, T, or C, were used as controls. Scaffold oligonucleotides (5′ GGTCTTCCACTGAATCATGGTCATAC 3′ and 5′ AAAACGACGGCCAGTGAATTGGACGC 3′) were used to ligate the 16mers into the M13 vector. The 19-mer of the sequence 5′GAAGACCTGGTAGCGCAGG 3′ was used to construct the “+3 competitor” for the CRAB assay.

DinB status of the constructed cell lines was confirmed using the upstream primer 5′ GATTATGGTGCTGACCAAAAGTGCG 3′ and the downstream primer 5′ CGCTGGCACTTAAGAGATATCCTGCGGG 3′. The M13 progeny DNA was amplified in the CRAB/REAP assays using the following: 5′ YCAGCTATGACCATGATTCAGTGGAAGAC 3′ (CRAP/REAP forward primer), 5′ YCAGGGTTTTCCCAGTCACGACGTTGTAA-3′ (CRAB reverse primer) and 5′ YTGTAAAACGACGGCCAGTGAATTGGACG 3′ (REAP reverse primer).

### Enzymes and chemicals

All restriction enzymes, T4 DNA Ligase, T4 DNA polymerase and their enzyme reaction buffers were from New England Biolabs. Shrimp alkaline phosphatase (SAP) was from Roche. P1 nuclease, 5-bromo-4-chloro-3-indolyl-beta-D-galactopyranoside (X-gal) and isopropyl β-D-1-thiogalactopyranoside (IPTG) were from Sigma Aldrich. T4 Polynucleotide kinase was from Affymetrix. Sephadex G-50 Fine resin was from Amersham Biosciences. Hydroxylapatite resin, 19∶1 acrylamide:bisacrylamide solution, and *N*,*N*,*N*′,*N*′-tetra-methyl-ethylenediamine (TEMED) were from Bio-Rad. Phenol:chloroform:isoamyl alcohol (25∶24∶1; pH 8) was from Invitrogen. ^32^P-γ-ATP was from Perkin Elmer. Non-radioactive ATP was from GE Healthcare Lifesciences.

### Construction of genomes

M13mp7(L2) phage single-stranded DNA starting material was isolated as described previously [57] (See Supporting Methods S1 in File S1). The oligonucleotides containing site-specific lesions were subsequently cloned in using reported methods [57]. Briefly, M13 single-stranded wild-type genomes were linearized with EcoRI, and scaffolds annealed to the ends. The16-mer oligonucleotide inserts were then annealed and ligated using T4 DNA ligase. The exonuclease activity of the T4 DNA polymerase was then used to digest the scaffolds. Finally, the constructed genomes were purified using phenol extraction and three TE washes in Microsep 100K spin dialysis columns. For details, see Supporting Methods S1 in File S1.

### Lesion bypass and mutagenesis assays

The relative bypass of each lesion was measured using the CRAB assay; mutational analysis was performed by using the REAP assay [57]. Briefly, the constructed viral genomes were first normalized using an established protocol [57]. Each lesion-containing genome was then mixed with the “+3” competitor genome in a 75∶25 ratio (ratio empirically determined, see Supporting Methods S1 in File S1) and then electroporated into *E. coli* strains of all combinations of AlkB and DinB proficiency and deficiency. After 6 h incubation at 37°C, the progeny phage were isolated and amplified by infecting SCS110 wild-type cells, to dilute out any lesion-containing genomes that did not electroporate and replicate in cells. Single-stranded M13 DNA was then isolated from the amplified progeny, using the M13 Qiaprep columns (Qiagen). The region of interest was then PCR amplified using the CRAB primers for the lesion bypass assay, or the REAP primers for the mutagenesis assay. The PCR products were subsequently digested with BbsI, HaeIII and radiolabeled to yield an 18-mer DNA fragment that contains at its 5′ end the specific site that initially contained the lesion of interest. The “+3” competitor genome was only amplified by the CRAB primers and yielded a 21-mer fragment. To quantitate the lesion bypass, the ratio between the intensities of the 18-mer and 21-mer fragments was determined and normalized to the ratio of the same bands for the unmodified “G” control, considered 100% bypass. To analyze the mutagenicity of a lesion, the radiolabeled 18-mer band was cut out from the gel and digested to single nucleotide monophosphates with nuclease P1. The nucleotides were then separated on PEI-TLC plates using a saturated solution of ammonium phosphate (pH = 5.8), and the radioactive signals quantitated using phosphorimagery. An approximately equimolar of GATC control genome mixture, which yielded four distinct TLC spots corresponding to the four normal nucleotides, was used as a mixture of standards. The detailed protocols for the CRAB and REAP assays are included in the Supporting Methods S1 in File S1.

## Results

### DinB Bypasses FF and HF Lesions *In Vivo*

The CRAB assay is a quantitative tool used to determine to what extent a given lesion blocks DNA replication *in vivo* (Figure 1B) [57]. In essence, a lesion-bearing genome is mixed with a nonlesion competitor genome in a specific input ratio and replicated in *E. coli* cells of a given repair/bypass genetic background. The output ratio of progeny phage from the lesion-bearing genome with respect to the nonlesion competitor genome indicates the relative amount of replication from the lesion-bearing genome. A decrease in the lesion:competitor output ratio signifies a lesion-induced replication block (i.e., lower bypass efficiency). The competitor genome, lacking a replication-inhibiting lesion, acts as an internal standard in this competitive assay. The CRAB assay was performed on the *N* ^2^-dG lesions (m2G, e2G, FF, and HF) in *E. coli* strains that capture all possible combinations of DinB and AlkB proficiency/deficiency (a total of 4 strains). The genomes of m3C, a good substrate for AlkB both *in vivo* and *in vitro* [53], and unmodified “G” were used as controls.

**Figure 2:**
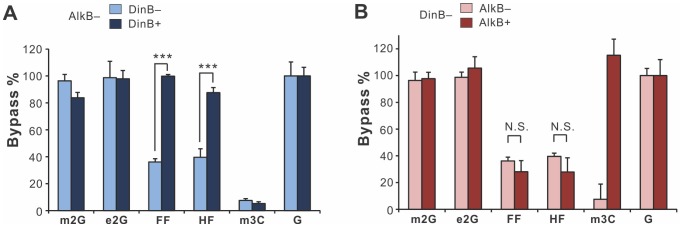
Bypass efficiency of m2G, e2G, FF, and HF as a function of DinB status (A) or AlkB status (B). M13 genomes containing the four *N* ^2^-alkylguanine lesions were constructed and normalized to one another before being combined with a competitor genome; genomes containing m3C and undamaged G were used as controls. Each mixture was transformed into the *E. coli* cell strains indicated at the top of each graph in triplicate, and bypass efficiencies were calculated by using the signal from the undamaged G genome mixture as 100% bypass; error bars represent one standard deviation. For the FF and HF lesions, the significance of the difference between two populations was tested using the Student's two-tailed *t* test. (*** indicates p-value <0.001, N.S. indicates not significant). All bypass data are summarized in Table S1 in File S1.

The results of the CRAB assay for toxicity are summarized in Figure 2 and Table S1 in File S1. To tease out the relative contribution of each genetic variable (DinB and AlkB) to the bypass efficiency of *N* ^2^-dG lesions, data were graphed in pairs in which one of the variables was kept constant. Based on the previously reported *in vitro* findings, it was hypothesized that both DinB and AlkB might act on *N* ^2^-dG lesions; therefore, the most relevant pair wise comparisons are those in which one of these enzymes is knocked out. To understand the effect of DinB on the *in vivo* bypass of *N* ^2^-dG lesions (independent of AlkB), the two AlkB^−^ strains were compared (AlkB^−^DinB^−^ vs. AlkB^−^DinB^+^) (Figure 2A). To understand the effect of AlkB (independent of DinB), the two DinB^−^ strains were compared (AlkB^−^DinB^−^ vs. AlkB^+^DinB^−^).

In the absence of DinB, the bulky lesions FF and HF are strong blocks to replication, with measured bypass efficiencies of only 36% and 40%, respectively (Figure 2A). However, the presence of DinB more than doubled the bypass efficiencies of FF and HF to ∼99% (p-value  = 0.0006) and ∼87% (p-value  = 0.0015), respectively (Figure 2A), thus greatly relieving the replication block. By contrast, the presence of AlkB did not change the bypass efficiencies of FF and HF; no significant difference in bypass was observed between the two DinB^−^ strains (Figure 2B). This finding was unexpected, given that AlkB is biochemically competent to repair the FF and HF lesions *in vitro* [59]. Possible reasons for AlkB's lack of effect on the bypass efficiencies of FF and HF are included in the discussion section.

The simple-alkyl lesions m2G and e2G were not significant replication blocks in the double mutant strain (AlkB^−^DinB^−^); the presence of DinB (Figure 2A), AlkB (Figure 2B) or both (Table S1 in File S1) did not significantly change the relative bypass of these lesions. From the point of view of this assay, these two modified guanines behave like a normal guanine, being virtually invisible to the replication machinery.

Consistent with previously published data [53], the m3C lesion was a good control for AlkB activity. In AlkB^−^ strains, m3C was a very strong block to replication (relative bypass ∼10%). The presence of DinB did not alleviate the toxicity of m3C (Figure 2A). However, in AlkB^+^ strains, the relative bypass of m3C jumped to ∼100%, consistent with the expectation that this lesion is efficiently repaired by AlkB, before being encountered by replicating polymerases.

### The *N^2^*-dG Lesions are Not Significantly Mutagenic in any Cell Strain

**Figure 3:**
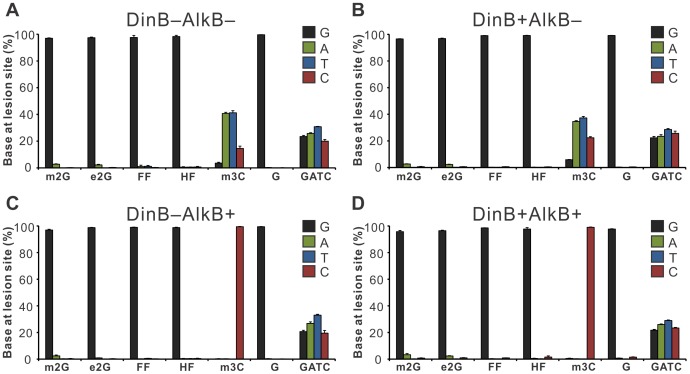
Mutagenesis of m2G, e2G, FF, and HF in the four strains of *E. coli* cells lacking or expressing DinB or AlkB. Each panel (**A** to **D**) corresponds to the *E.coli* strain indicated at the top of the graph. Genomes containing m3C, undamaged G, and an approximately equimolar mixture of unmodified G/A/T/C bases at the site of inquiry (denoted as GATC) were used as controls. Genomes containing the lesions of interest were transfected into *E. coli* in triplicate. The percentage of G, A, T, and C at the lesion site reveals the mutagenicity of the lesions, with error bars representing one standard deviation. All mutagenesis data are summarized in Table S2 in File S1.

The REAP assay determines the mutation frequency and mutation type after the lesion of interest has been processed by the intracellular replication machinery [57]. The REAP assay was performed on all of the *N* ^2^-dG lesions, the control m3C, unmodified G, and an approximately equimolar mixture of genomes carrying unmodified ‘G’, ‘A’, ‘T’, and ‘C’ bases at the lesion site (denoted as GATC, Figure 3).

One could not predict a priori whether or not these lesions would be mutagenic *in vivo*, but the results clearly show that none of the *N^2^*-dG lesions are significantly mutagenic in the presence or absence of DinB or AlkB (Figure 3 and Table S2 in File S1). HF and FF show mutation frequencies of ∼1% regardless of DinB status, which is essentially the same as the mutation frequency we detected for the control genome having a normal G at the lesion site. m2G and e2G also show a small non-G signal of 1 to 4% in all cell strains (Table S2 in File S1), but this mutation frequency is not statistically different from the control genome baseline.

## Discussion

### The Effect of DinB on *N* ^2^-dG Lesions

In this study, the role of the *E. coli* DNA TLS polymerase DinB in bypassing a spectrum of *N^2^*-alkylguanine lesions *in vivo* was investigated. In 2006, it was discovered that *in vitro*, the TLS polymerase DinB efficiently bypasses the FF adduct (Figure 1A), a homolog of the major adduct formed by the reaction of NFZ with guanine [4]. Catalytically, DinB is about 15-fold more proficient at inserting a cytosine opposite the FF adduct than opposite undamaged guanine [4]. Additionally, due to its increased affinity for dCTP, DinB is 25-fold more efficient at extending beyond a cytosine opposite the FF lesion than opposite guanine [40]. The current study is the first *in vivo* quantitative analysis of the mutagenic and toxic properties of the FF lesion and its saturated homolog HF, as a function of the DinB genotype of the cell. The key findings of this study are: 1) FF and HF are strong blocks to replication when introduced in DinB^−^ cells on a single-stranded vector; 2) The replication inhibition caused by FF and HF is substantially alleviated by the presence of DinB *in vivo*, further supporting the role of DinB in *N* ^2^-alkylguanine lesion bypass observed previously *in vitro* [4]; 3) The lesion bypass occurs in an error-free manner, as the correct base (cytosine) is always inserted opposite the guanine lesions by DinB. This last finding is also in concordance with previously published *in vitro* bypass results [4], [40]. Taken together with the *in vitro* data available for DinB and its homologs, the current study suggests that these Y-family polymerases bypass bulky *N* ^2^-guanine adducts, such as the one formed by NFZ, in an error-free manner, *in vivo*. Given that NFZ is an antibiotic, DinB may be an important biochemical shield evolved for the defense of *E. coli* against certain types of ‘chemical warfare’ from other species. This finding is also consistent with the proposed role of DinB in transcription-coupled translesion synthesis across *N* ^2^-dG lesions formed by NFZ [60]. It is worth noting that, while FF and HF are strong blocks to replication in the absence of DinB, the level of bypass detected in the DinB^−^ cells (28 to 40%) was higher than that seen for the concurrently run positive control m3C (∼10%) or other alkyl lesions (i.e., m1G or m3T) tested previously in AlkB-negative cells [53]. One possible explanation is that there might be other bypass/repair mechanisms at play (i.e., Pol V) that assist with lesion tolerance in the absence of DinB to the extent observed in this study. While it has been proposed that nucleotide excision repair (and not TLS) might be the primary repair pathway that deals with NFZ-induced damage [61], that pathway requires a double-stranded DNA context, which is obviated by our experimental system that utilizes single-stranded M13 genomes.

In contrast to the bulky FF and HF lesions, the small *N* ^2^-alkylguanine lesions m2G and e2G were neither replication blocks nor were they mutagenic in any of the *E. coli* cells tested. Since no replication inhibition was seen in DinB^−^ cells, the presence of DinB did not change the bypass efficiencies of m2G and e2G; in fact, there is no evidence that DinB was actually recruited at the replication fork, when m2G or e2G lesions were encountered. This *in vivo* result under physiological conditions is in contrast with what has been observed for e2G in *in vitro* assays with other Y-family and replicative polymerases [62]–[64]. It could be that there is another enzyme or enzymes that preferentially and efficiently repairs or bypasses these lesions such that the supplementary role of DinB in the bypass of m2G and e2G is overshadowed beyond the detection limit of our assay.

One possible explanation for the non-toxic phenotype of FF and HF seen in DinB+ cells is that DinB can tolerate the *N^2^*-alkyl dG lesions. These lesions can occupy the minor groove of DNA [65] and interfere with polymerase-minor groove interactions [66]–[68], should the alkyl group swivel near the N3 atom of guanine. Several B-family polymerases are known to have a conserved motif that scans the DNA minor groove for lesions and misincorporations [69], which is lacking in the Y-family DNA polymerases. It is speculated that this may be the case for the Y-family mammalian pol κ, as deduced from X-ray crystal structure studies of the catalytic core of the polymerase with a primer-template DNA and an incoming nucleotide; the structure reveals the lack of a “steric gate” in scanning the minor groove at the primer-template junction [70]. It is proposed that DinB can accommodate minor groove lesions to enable bypass with correct base pairing, even for lesions such as BaP [45], containing alkyl groups much bulkier than those used in the current study.

The FF and HF lesions, while they are strong replication blocks, are not mutagenic in any repair/bypass background; these results can be explained by the availability of a hydrogen atom at the N2 position of guanine and the possibility of free rotation around guanine's exocyclic nitrogen-carbon bond. This free rotation can generate a guanine-like Watson-Crick hydrogen bonding pattern with cytosine. In addition, free rotation around the carbon-nitrogen bond would enable the extraneous alkyl group, irrespective of size, to swivel away from the base pairing face of guanine into the minor groove, thus alleviating steric hindrance caused by attachments to the N2 position. Similar freely rotating small alkyl modifications, such as *N* ^6^-methyladenine and *N* ^4^-methylcytosine, are very well tolerated and even utilized as DNA replication biomarkers in prokaryotic cells [71]. Alternative mechanisms to explain the correct base pairing of *N^2^*-dG lesions with cytosine, such as ‘wobble’ base pairing [63], or Hoogsteen base pairing [62], [72] have been proposed or observed. However, for an *N* ^2^-dG lesion to pair correctly with a cytosine using either mechanism, the cytosine base has to be in its protonated form (for Hoogsteen base pairing), or its imine tautomeric form (for wobble base pairing), which is rarely observed in duplex DNA under physiological conditions.

### The Effect of AlkB on *N* ^2^-dG Lesions

A number of *N* ^2^-dG lesions were tested as possible substrates for AlkB, in line with the theme of this study on the cellular processing of *N* ^2^-guanine DNA lesions. While all the four *N* ^2^-dG lesions studied are repaired by AlkB *in vitro* [59], the results from this study show that AlkB does not have a discernible effect on either the polymerase bypass or mutagenicity of these lesions *in vivo*. FF and HF are replication blocks in the absence of DinB (Figure 2A and Table S1 in File S1). While the presence of DinB alleviates the replication inhibition, AlkB has no significant effect on the bypass efficiencies of FF and HF lesions in DinB^−^ cells (Figure 2B and Table S1 in File S1). There are two possible explanations for these experimental observations: 1) Given AlkB's low cellular concentration (2 molecules/cell) [73], it does not effectively repair the bulky HF and FF lesions *in vivo*, before they are encountered by the replication machinery; or 2) AlkB does perform the initial oxidation step on these lesions, but the subsequent intermediates, (such as FF-2H and HO-HF, described in our previous paper [59]), may be long-lived and equally strong replication blocks. By contrast, the smaller *N* ^2^-dG lesions (m2G and e2G) were not toxic or mutagenic in any of the four cell strains studied (Figures 2 and 3). This suggests that at least in AlkB^−^ cells, a mechanism of tolerance of small minor groove lesions by replicative polymerases is operating; as mentioned above, these lesions may rotate into the minor groove during replication [59], [70]. However, effective repair of m2G or e2G by AlkB *in vivo* may still occur in the AlkB^+^ cell strains, supplementing the free rotation mechanism.

In conclusion, this work demonstrates that inside living cells, DNA adduct bypass by DinB is the mechanism of choice to overcome the deleterious consequences of bulky *N* ^2^-dG adducts, such as FF and HF. While we have shown that AlkB can repair these lesions and the simpler m2G and e2G adducts *in vitro*, the AlkB effect on FF and HF *in vivo* is not significant, possibly because the repair intermediates are also strong replication blocks, long-lived and not cleared efficiently before encountering replication machinery. Our study highlights the fact that even though multiple DNA repair or tolerance pathways can act on *N* ^2^-alkylguanine DNA lesions *in vitro*, one pathway, lesion bypass, is the preferred mechanism for maintaining genomic integrity *in vivo*.

## Supporting Information

File S1 Supporting Methods S1, Supporting Tables S1–S2 and Supporting References.
(PDF)
