# Supplementary material for: A Chemical Genetics Analysis of the Roles of Bypass Polymerase DinB and DNA Repair Protein AlkB in Processing N 2-Alkylguanine Lesions In Vivo
Source: PLoS One. 2014 Apr 14;9(4):e94716. doi: 10.1371/journal.pone.0094716 (PMC3986394; doi:10.1371/journal.pone.0094716)
Supplement: File S1 — Supporting Methods S1, Supporting Tables S1–S2 and Supporting References. (PDF) [file pone.0094716.s001.pdf]

SUPPORTING INFORMATION FILE S1

A Chemical Genetics Analysis of the Roles of Bypass Polymerase DinB and DNA Repair Protein AlkB in Processing N^2-Alkylguanine Lesions *In Vivo*

Nidhi Shrivastav, Bogdan I. Fedeles, Deyu Li, James C. Delaney, Lauren E. Frick, James J. Foti,

Graham C. Walker, and John M. Essigmann*

*jessig@mit.edu

TABLE OF CONTENTS

Supporting Method S1

Supporting Tables

Table S1. Bypass efficiencies of N^2-dG lesions as determined by the CRAB assay.

Table S2. Mutagenicity of the N^2-dG lesions as determined by the REAP assay.

Supporting References

Supporting Methods S1

The REAP and CRAB assays described here have been adapted from the work of Delaney and Essigmann [1]. Please refer to the original method for more detail and clarification.

Double agar overlay plaque method for phage analysis

The double agar overlay method[1] was used for enumerating initial electroporation events as well as phage titers to ensure statistical robustness, but not for mutational analyses. Briefly, 10 ml of 2 x YT media was inoculated with 2 ml of a saturated overnight culture of NR9050 and grown for 1 h at 37 °C with aeration. Three hundred µl of this culture was mixed with 10 µl IPTG (24 mg/ml), 25 µl of 1 % thiamine, and 40 µl X-Gal (40 mg/ml in DMF), added to 2.5 ml of top agar maintained in a molten state at 52 °C to which electroporated cells or appropriate dilutions of supernatants containing phage particles in LB were immediately mixed and poured evenly onto B-broth plates. After a 10 min incubation at room temperature (to allow the top agar to solidify), the plates were incubated overnight at 37 °C to obtain dark blue, light blue, or clear plaques.

M13 phage DNA

M13mp7(L2) phage single-stranded DNA starting material was isolated as follows. Various dilutions of a previous stock of M13 phage supernatant were plated on a lawn of *E. coli* cells using the double agar overlay method to obtain phage plaques. A well-isolated plaque was plugged using a sterile Pasteur pipette and vortexed in 1 ml LB, 200 µl of which was used to make a starter culture (grown overnight) by mixing with 10 µl of an overnight saturated culture of GW5100 cells in 10 ml LB. One milliliter of this phage starter culture was then used to inoculate GW5100 cells, which had been grown using 500 µl of an overnight saturated culture in 250 ml of fresh 2 x YT medium for 2 h at 37 °C and shaken at 275 rpm. The inoculated culture was grown further for 8 h at 37 °C with aeration, after which the cells were pelleted and discarded. The phage was precipitated from the supernatant by making it 4% PEG 8000 MW and 0.5 M NaCl. After overnight precipation at 4 °C, the phage were pelleted, resuspended in 5 ml TE pH 8, and extracted four times with 3 ml 25:24:1 phenol:chloroform:isoamyl alcohol. The aqueous phase was passed through a 0.5 g hydroxylapatite column (BioRad), washed with 5 ml TE, and eluted in 1 ml fractions with 12 ml of 0.16 M phosphate buffer. The DNA-containing fractions were identified by spotting on an agarose plate containing ethidium bromide. The phosphate buffer in those fractions was then exchanged for TE by three washes in Microsep 100K spin dialysis columns (Pall Lifesciences). The DNA obtained was at a yield of ≥ 1 pmol/ml of 2 x YT large culture, and was stored at -20 °C until further use.

Construction of genomes

The multiple cloning site in single-stranded M13mp7(L2) is designed to form a hairpin structure that contains a functional *Eco*RI site. Twenty picomoles of M13 single-stranded DNA were linearized by incubation with 40 U of *Eco*RI for 8 h at 23 °C. Scaffolds (25 pmol in 1 µl each) were annealed to the ends of the linearized genome by incubation at 50 °C for 5 min followed

by cooling to 0 °C over 50 min. In addition, 30 pmol of each 16-mer oligonucleotide insert was 5' phosphorylated by 15 U of T4 PNK, supplemented with 1x T4 PNK buffer, 1 mM ATP, and 5 mM DTT and incubated at 37 °C for 1 h. The linearized genome was mixed and subsequently ligated with the phosphorylated oligonucleotide for 8 h at 16 °C in a reaction volume of 75 µl containing 1 mM ATP, 10 mM DTT, 25 ug/ml BSA, and 800 U T4 DNA ligase. To degrade scaffolds and unligated oligonucleotides, the ligation mixture was treated with 0.25 U/µl T4 DNA polymerase (containing an exonuclease domain) for 4 h at 37 °C. Finally, the reaction volume was brought up to 110 µl with water and extracted once with 100 µl 25:24:1 phenol:chloroform:isoamyl alcohol. The aqueous phase was purified by three TE (pH 8) washes in Microsep 100K spin dialysis columns to remove any residual phenol and salts. Recovery yields of 30-45% were obtained.

Genome validation and normalization
Prior to proceeding with the bypass and mutagenicity assays, the incorporation of lesion-containing oligonucleotides was confirmed and the relative concentration of the constructed genomes was determined and normalized using the following procedure: A 10-fold molar excess of scaffolds (previously used in constructing the genomes) were annealed to ~0.35 pmol of genomes in 5 µl. The genomes were cleaved with 10 U of *Hin*FI and the resulting 5' end dephosphorylated with 1 U of shrimp alkaline phosphatase in a reaction volume of 8 µl with incubation at 37 °C for 1 h followed by phosphatase inactivation at 80 °C for 5 min and cooling down to 20 °C @ 0.2 °C /s. The 5' ends were then labeled with 1.66 pmol of ^{32}P-γ-ATP (6000 Ci/mmol) in a total reaction volume of 12 µl containing 1 x buffer 2, 5 mM DTT, 150 pmol cold ATP, 5 U T4 PNK, and 10 U *Hae*III, with incubation at 37 °C for 1 h. The reaction was stopped by the addition of 12 µl 2 x formamide loading buffer, and the products were resolved using 20% PAGE until the xylene cyanol dye migrated a distance of 12 cm. The bands corresponding to fully-ligated genomes were then quantified using phosphorimagery and normalized with respect to one another. The genomes were then diluted with water such that all the genomes were at the same final concentration. The band generated from the competitor genome was used as a marker.

Post-normalization, a test electroporation was performed in HK81 electrocompetent cells using the control genome mixed in different ratios with the competitor genome. The results of the test electroporation were determined by plating the cells immediately after electroporation using the phage-overlay method to yield a dark blue (control): light blue (competitor) plaque count ratio. The ratio that yielded a 75:25 dark blue:light blue phage count was selected as the formulation ratio for the bypass assay of the lesion-containing genomes.

Preparation of electrocompetent cells
Three baffled flasks containing 150 ml LB medium each were inoculated with 1.5 ml of a saturated overnight culture of the strain to be transformed. The cultures were incubated at 37 °C and shaken at 275 rpm for ~2.5 h until the cultures reached early log phase, as measured by OD_{600} of ~0.5. The cell were then pelleted by centrifugation at 9500 rpm (Sorvall GSA rotor), resuspended in 1 ml cold sterile water, and pooled to a final volume of 175 ml in cold sterile water, which was pelleted and resuspended in 175 mL water a total of three times. The final

resuspension was in 4.8 ml 10% glycerol to obtain a final volume of 6 ml electrocompetent cells, which were then aliquoted and stored at -80 °C prior to use.

CRAB assay

Genomes containing the lesions were mixed at a 75:25 ratio of genome:competitor, and 6 µl were mixed with 100 µl competent cells and electroporated in triplicate in a 2 mm-gap cuvette using 2.5 kV and 125 Ω. The cells were immediately transferred to 10 ml LB and an aliquot of the freshly electroporated cells was immediately plated using the agar overlay method to ensure that a minimum of 10^5 independent initial electroporation events occurred in 10 ml of culture. The cells were then grown for 6 h at 37 °C with aeration to amplify progeny phage. The supernatants of the 6 h cultures were retained and plated using the agar overlay method to confirm 10^4 -fold amplification in the progeny phage titer. Another round of amplification was performed in order to dilute the DNA from the residual genomes that did not get electroporated into cells; thus, the subsequent PCR step utilized only the DNA from the progeny phage from genomes that entered the *E. coli* cells. The amplification was done by infecting 10 µl of an overnight culture of SCS110 cells with 100 µl of the 6 h supernatants in 10 ml LB and incubating for 7 h at 37 °C with aeration, after which the supernatant was retained. Single-stranded M13 progeny phage DNA was isolated from 0.7 ml of supernatant using a QIAprep Spin M13 Kit with final DNA suspension in 100 µl elution buffer. CRAB forward and reverse primers were used to amplify the region of interest from 10 µl per QIAprep elution sample in a total volume of 25 µl using 1.25 U *Pfu Turbo* DNA polymerase, 25 mM of each dNTP, and 10 x *Pfu Turbo* buffer. The PCR program started by denaturing at 94 °C for 5 min, then cycled 30 times at 94 °C for 30 s, 67 °C for 1 min, and 72 °C for 1 min, and finally extended for 5 min at 72 °C. The volume was then made up to 110 µl using water and extracted once using 25:24:1 phenol:chloroform:isoamyl alcohol to destroy the DNA polymerase (including the exonuclease domain). The aqueous phase was passed through a Sephadex G-50 Fine resin spin column to remove any remaining dNTPs and traces of phenol.

The purified PCR product was then treated with *Bbs*I (1.5 U for 4 µl of sample in a total volume of 6 µl) and shrimp alkaline phosphatase (0.3 U) by incubating at 37 °C for 4 h, heating to 80 °C for 5 min to inactivate the phosphatase, and cooling to 20 °C @ 0.2 °C /s. The 5' ends were then labeled in a total volume of 8 µl with a mixture of non-radioactive ATP (20 pmol), γ-^{32}P-ATP (1.66 pmol of 10 uCi/µl at 6000 Ci/mmol), and 5 U of Optikinase/T4 PNK, with incubation at 37 °C for 15 min, followed by 65 °C for 20 min to inactivate the kinase and cooling to 23 °C @ 0.1 °C /s. The labeled product was then trimmed by *Hae*III (10 U in a final volume of 10 µl) at 37 °C for 2 h, followed by addition of 10 µl 2 x formamide loading dye, which quenched the reaction. The samples were then loaded onto a 20% denaturing gel and run for ~3.5 h at 550 V, until the xylene cyanol dye migrated 10.5 cm. The gel was then exposed to a phosphorimager screen and quantified using a Storm 840 scanner. Band intensities were quantified using ImageQuant 5.2 (Molecular Dynamics) or ImageJ software (National Institutes of Health); lesion bypass was then calculated as the percentage ratio of the intensity of the 18-mer band (lesion signal) to the intensity of the 21-mer band (competitor signal).

REAP assay

The REAP assay methodology is identical to the CRAB assay except for the PCR primers. The primers used for the REAP assay span both the vector as well as the insert at each end, thereby effecting a selective amplification of DNA from only the progeny phage resulting from the lesion-carrying genomes. Following electrophoresis, 20% denaturing gel analysis showed no frameshift mutagenesis, and the 18-mer bands were excised from the gel, and crushed and soaked overnight in 200 µl water. After desalting with Sephadex G-50 Fine resin spin columns, the samples were lyophilized overnight to dryness, resuspended in 5 µl containing 1 µg P1 Nuclease in 30 mM sodium acetate and 100 mM zinc chloride, and incubated at 50 °C for 1 h. One µl of each sample was then spotted onto water-washed PEI-TLC plates, and ^{32}P-dNMPs were separated using 200 ml of a saturated solution of $(NH_4)_2HPO_4$ adjusted to pH 5.8. After 12 h of development, the TLC plates were air-dried and quantified using phosphorimagery.

Supporting Tables

Table S1. Bypass efficiencies (reported as a percentage relative to unmodified G) of N^2-dG lesions as determined by the CRAB assay. The data tabulated below are shown in Figure 3.

Lesion	DinB+/AlkB+ cells		DinB+/AlkB− cells		DinB−/AlkB+ cells		DinB−/AlkB− cells	
	Avg.	Std. Dev.	Avg.	Std. Dev.	Avg.	Std. Dev.	Avg.	Std. Dev.
**m2G**	90.5	4.7	83.8	4.0	97.7	6.2	96.4	4.8
**e2G**	99.8	8.5	97.9	6.2	105.6	3.9	98.7	12.2
**FF**	101.9	8.4	99.9	1.2	28.1	2.9	36.1	2.4
**HF**	91.8	10.6	87.6	3.8	27.9	2.4	39.6	6.3
**m3C**	98.2	12.1	5.2	1.3	115.2	11.4	7.5	1.4
**G**	100.0	11.9	100.0	6.4	100.0	5.4	100.0	10.4

Table S2. Mutagenicity of the N^2-dG lesions as determined by the REAP assay. The data tabulated below are shown in Figure 4.

**(a) DinB+/AlkB+ cells**								
**Lesion/base**	**Average**				**Standard deviation**			
	**% G**	**% A**	**% T**	**% C**	**% G**	**% A**	**% T**	**% C**
**m2G**	95.7	3.3	0.2	0.8	0.9	0.9	0.0	0.2
**e2G**	96.5	2.3	0.2	1.0	0.3	0.2	0.0	0.1
**FF**	98.5	0.3	0.2	1.0	0.1	0.1	0.0	0.1
**HF**	97.7	0.5	0.3	1.5	1.1	0.2	0.1	0.9
**m3C**	0.5	0.3	0.2	99.0	0.2	0.0	0.0	0.2
**G**	97.7	0.7	0.1	1.5	0.3	0.1	0.0	0.3
**GATC**	21.7	26.0	29.0	23.3	0.6	0.5	0.4	0.4
**(b) DinB+/AlkB− cells**								
**Lesion/base**	**Average**				**Standard deviation**			
	**% G**	**% A**	**% T**	**% C**	**% G**	**% A**	**% T**	**% C**
**m2G**	96.6	2.7	0.1	0.6	0.1	0.1	0.0	0.2
**e2G**	96.9	2.3	0.2	0.6	0.3	0.2	0.0	0.1
**FF**	99.0	0.2	0.2	0.6	0.1	0.1	0.0	0.0
**HF**	99.1	0.2	0.2	0.5	0.2	0.1	0.0	0.0
**m3C**	5.9	34.5	37.2	22.4	0.2	0.7	1.2	0.8
**G**	99.1	0.3	0.1	0.5	0.1	0.0	0.0	0.1
**GATC**	22.3	23.4	28.6	25.7	1.0	1.2	0.7	1.7
**(c) DinB−/AlkB+ cells**								
**Lesion/base**	**Average**				**Standard deviation**			
	**% G**	**% A**	**% T**	**% C**	**% G**	**% A**	**% T**	**% C**
**m2G**	96.9	2.5	0.2	0.4	0.6	0.6	0.1	0.1
**e2G**	98.7	0.9	0.1	0.3	0.2	0.1	0.0	0.1
**FF**	99.0	0.2	0.6	0.2	0.1	0.0	0.0	0.1
**HF**	98.7	0.4	0.4	0.5	0.3	0.2	0.0	0.2
**m3C**	0.3	0.2	0.1	99.4	0.1	0.1	0.0	0.2
**G**	99.4	0.4	0.1	0.1	0.1	0.1	0.0	0.1
**GATC**	20.7	26.8	33.0	19.5	0.9	1.2	0.7	2.0
**(d) DinB−/AlkB− cells**								
**Lesion/base**	**Average**				**Standard deviation**			
	**% G**	**% A**	**% T**	**% C**	**% G**	**% A**	**% T**	**% C**
**m2G**	97.0	2.7	0.1	0.2	0.3	0.3	0.0	0.0
**e2G**	97.4	2.2	0.1	0.3	0.4	0.5	0.0	0.1
**FF**	97.5	1.0	1.2	0.3	1.6	1.0	0.7	0.1
**HF**	98.3	0.5	0.6	0.6	0.8	0.3	0.1	0.4
**m3C**	3.6	40.7	41.2	14.5	0.6	0.8	1.6	1.7
**G**	99.6	0.3	0.0	0.1	0.1	0.1	0.0	0.1
**GATC**	23.4	25.8	30.8	20.0	0.8	0.5	0.3	1.3

Supporting References

1. Delaney JC, Essigmann JM (2006) Assays for determining lesion bypass efficiency and mutagenicity of site-specific DNA lesions in vivo. Meth Enzymol 408: 1–15. doi:10.1016/S0076-6879(06)08001-3.

2. Frick LE (2007) The versatile E. coli adaptive response protein AlkB mitigates toxicity and mutagenicity of etheno-, ethano-, and methyl-modified bases in vivo. Massachusetts Institute of Technology: PhD Thesis. Available: http://dspace.mit.edu/handle/1721.1/42382. Accessed 5 December 2012.

3. Frick LE, Delaney JC, Wong C, Drennan CL, Essigmann JM (2007) Alleviation of 1,N6-ethanoadenine genotoxicity by the Escherichia coli adaptive response protein AlkB. Proc Natl Acad Sci USA 104: 755–760. doi:10.1073/pnas.0607377104.

4. Delaney JC, Essigmann JM (2004) Mutagenesis, genotoxicity, and repair of 1-methyladenine, 3-alkylcytosines, 1-methylguanine, and 3-methylthymine in alkB Escherichia coli. Proc Natl Acad Sci USA 101: 14051–14056. doi:10.1073/pnas.0403489101.

5. Jarosz DF, Godoy VG, Delaney JC, Essigmann JM, Walker GC (2006) A single amino acid governs enhanced activity of DinB DNA polymerases on damaged templates. Nature 439: 225–228. doi:10.1038/nature04318.
